# Integrating Datasets on Public Health and Clinical Aspects of Sickle Cell Disease for Effective Community-Based Research and Practice

**DOI:** 10.3390/diseases8040039

**Published:** 2020-10-26

**Authors:** Raphael D. Isokpehi, Chomel P. Johnson, Ashley N. Tucker, Aakriti Gautam, Taylor J. Brooks, Matilda O. Johnson, Thometta Cozart, Deanna J. Wathington

**Affiliations:** 1 Center for Trans-Disciplinary Data Analytics, Bethune-Cookman University, Daytona Beach, FL 32114, USA; ashley.n.tucker@students.cookman.edu (A.N.T.); aakriti.gautam@students.cookman.edu (A.G.); taylor.j.brooks@students.cookman.edu (T.J.B.); 2 Master of Public Health Program, Bethune-Cookman University, Daytona Beach, FL 32114, USA; chomel.p.johnson@students.cookman.edu (C.P.J.); johnsonma@cookman.edu (M.O.J.); cozartt@cookman.edu (T.C.); wathingtond@cookman.edu (D.J.W.); 3 Health Equity Internship Program, Association of State Public Health Nutritionists, P.O. Box 37094, Tucson, AZ 85740, USA; 4 Department of Public Health and Health Equity, Bethune-Cookman University, Daytona Beach, FL 32114, USA

**Keywords:** community-based health, clinical manifestations, data analytics, evidence-based practice, health promotion, interactive analytics, public health, sickle cell disease, visual analytics

## Abstract

Sickle cell disease (SCD) is a genetic disease that has multiple aspects including public health and clinical aspects. The goals of the research study were to (1) understand the public health aspects of sickle cell disease, and (2) understand the overlap between public health aspects and clinical aspects that can inform research and practice beneficial to stakeholders in sickle cell disease management. The approach involved the construction of datasets from textual data sources produced by experts on sickle cell disease including from landmark publications published in 2020 on sickle cell disease in the United States. The interactive analytics of the integrated datasets that we produced identified that community-based approaches are common to both public health and clinical aspects of sickle cell disease. An interactive visualization that we produced can aid the understanding of the alignment of governmental organizations to recommendations for addressing sickle cell disease in the United States. From a global perspective, the interactive analytics of the integrated datasets can support the knowledge transfer stage of the SICKLE recommendations (Skills transfer, Increasing self-efficacy, Coordination, Knowledge transfer, Linking to adult services, and Evaluating readiness) for effective pediatric to adult transition care for patients with sickle cell disease. Considering the increased digital transformations resulting from the COVID-19 pandemic, the constructed datasets from expert recommendations can be integrated within remote digital platforms that expand access to care for individuals living with sickle cell disease. Finally, the interactive analytics of integrated expert recommendations on sickle cell disease management can support individual and team expertise for effective community-based research and practice.

## 1. Introduction

Sickle cell disease (SCD) is a group of life-threatening inherited disorders (sickle cell anemia, sickle cell-hemoglobin C disease, and the sickle cell-β-thalassemias) caused by changes in the gene that encodes hemoglobin subunit beta, a component of the oxygen carrying hemoglobin protein of the red blood cells [1,2]. The most common genotype of sickle cell disease is sickle cell anemia (SCA), where the gene for the changed protein form is inherited from both parents [3]. Sickle cell anemia has a high prevalence in people of African ancestry [4]. Although Sickle Cell Disease (SCD) is a monogenic disease, there is a heterogeneity in clinical manifestations including stroke, pain, renal failure and pulmonary hypertension [5,6]. In the United States, the statistics on sickle cell disease include: (1) sickle cell disease affects approximately 100,000 Americans; (2) sickle cell disease occurs among about (a) 1 out of every 365 Black or African-American births, and (b) 1 out of every 16,300 Hispanic-American births; and (3) about 1 in 13 Black or African-American babies is born with sickle cell trait (SCT) [7].

**Figure 1:**
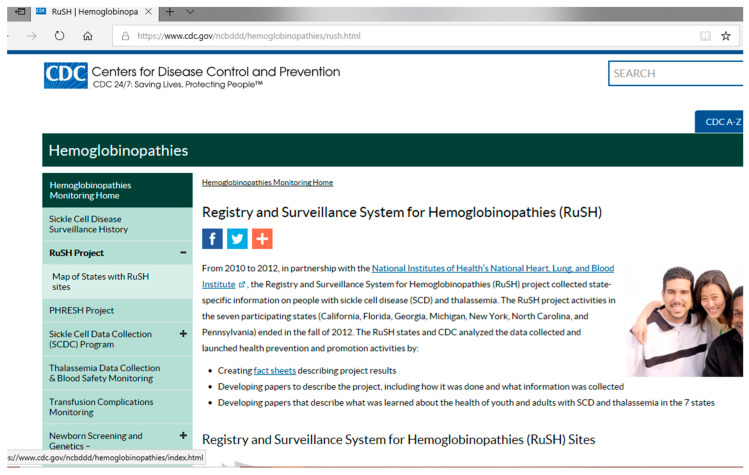
Website of the Registry and Surveillance System for Hemoglobinopathies (RuSH). The web page includes listing of the public health programs on hemoglobinopathies at the Centers for Disease Control and Prevention.

The primary goal of the research study reported here is to understand the public health aspects of sickle cell disease. A secondary goal is to understand the overlap between public health aspects and clinical aspects that can inform research and practice beneficial to stakeholders of sickle cell disease management. The public health aspects of interest are prevalence, impact on health, health costs, and health promotion. In the United States, programs with a focus on public health aspects of sickle cell available through the Centers for Disease Control and Prevention include: (1) the Registry and Surveillance System for Hemoglobinopathies (RuSH); (2) Public Health Research, Epidemiology, and Surveillance for Hemoglobinopathies (PHRESH) and (3) the Sickle Cell Data Collection (SCDC) Program [8] (Figure 1).

**Figure 2:**
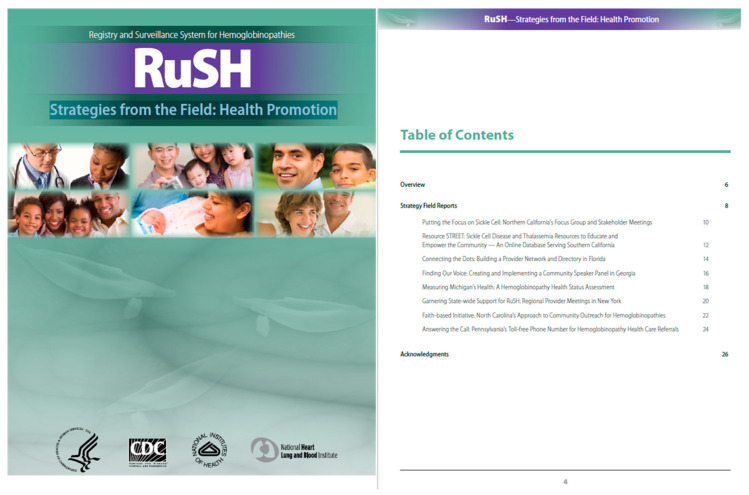
Report on health promotion strategies for sickle cell diseases from eight sites in the United States. The location of the participating sites in the report are California, Florida, Georgia, Michigan, New York, North Carolina and Pennsylvania.

**Figure 3:**
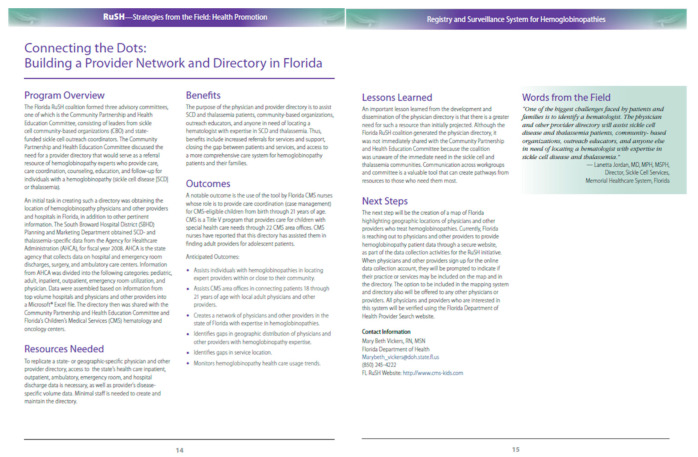
Data source for interactive analytics of public health aspects of sickle cell disease. Each report from a RuSH site included the program overview, resources needed, benefits, outcomes, lessons learned and next steps.

These public health projects on sickle cell disease provide data and reports that can be the basis for understanding the public health aspects of sickle cell disease. We have selected to focus our investigation on the RuSH project because it is the first in the series of surveillance (monitoring) projects that started in 2010 by the Centers for Disease Control and Prevention. Additionally, the report (health promotion and data collection) is from seven participating states, making it more comprehensive than the PHRESH (three states) and SCDC (two states). The RuSH report on health promotion contains information on health promotion strategies that are state-specific, population-based, and take into account health status, treatment and healthcare utilization (Figure 2) [9,10]. A challenge to using the report to understand public health aspects is that the information is presented as individual project reports. Therefore, an integration of the sections (an example of textual datasets) in the 2012 Registry and Surveillance System for Hemoglobinopathies (RuSH) report on health promotion is one of the goals of our study. The sections are Program Overview; Resources Needed; Benefits; Outcomes; and Lessons Learned (Figure 3).

**Figure 4:**
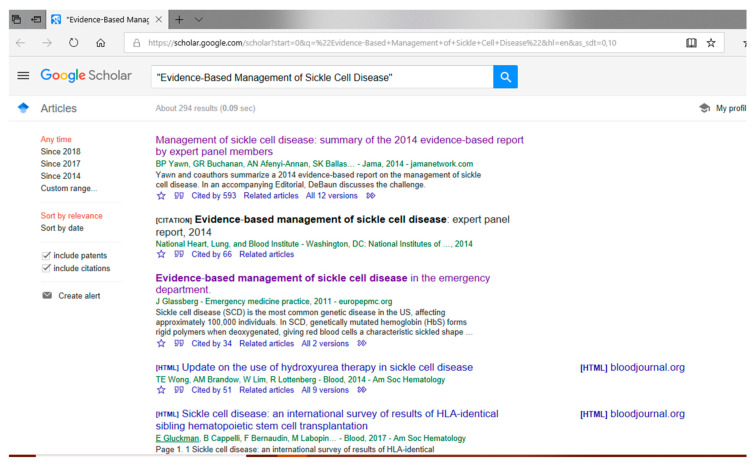
Search in Google Scholar for citations of the data source for interactive analytics of public health aspects of sickle cell disease.

In September 2014, the National Heart, Lung and Blood Institute (NHLBI) of the U.S. National Institutes of Health (NIH) published an expert panel report that provides a synthesis of available scientific evidence on sickle cell disease and offers guidance to busy primary care clinicians [6]. This NHLBI expert panel report on sickle cell disease management (also referred here as the 2014 NHLBI SCD report or 2014 NHLBI report) consists of seven chapters and presents recommendations on the management of clinical aspects of sickle cell disease including managing acute and chronic complications. We reason that the citation context of the 2014 NHLBI SCD report in journal articles could contain public health aspects (Figure 4). Additionally, comparing textual data from the 2012 RuSH report and 2014 NHLBI SCD report will reveal themes for sickle cell disease collaboration between clinical and public health professionals.

In 2019 and 2020, additional expect panel recommendations on the clinical and public health aspects of sickle cell disease were published. The American Society of Hematology (ASH) published sickle cell disease clinical practice recommendations on cardiopulmonary and kidney disease, transfusion support, cerebrovascular disease, and the management of acute and chronic pain [11,12,13,14]. The clinical aspects in the 2019–2020 ASH SCD guidelines overlap with the 2014 NHLBI SCD report presenting opportunities for comparisons and integrations of the expert panel recommendations.

In September 2020, the National Academies of Sciences, Engineering, and Medicine (NASEM) published a consensus study report titled “Addressing Sickle Cell Disease: A Strategic Plan and Blueprint for Action”, which includes eight overarching strategies and associated recommendations to accomplish a strategic vision of “long, healthy, productive lives for those living with sickle cell disease and those with sickle cell trait” [15]. Among the 2020 NASEM SCD report recommendations are strategic partnerships that include community-based organizations and patient advocates. For example, Strategy E of the 2020 NASEM SCD report aims to “Improve sickle cell disease awareness and strengthen advocacy efforts through targeted education and strategic partnerships among the U.S. Department of Health and Human Services, health care providers, advocacy groups and community-based organizations, professional associations, and other key stakeholders (e.g., media and state health departments).”

These expert panel or consensus study reports, which encode expert knowledge on sickle cell disease, are voluminous, professionally-collected textual data sources that are suitable for constructing integrated datasets from multi-faceted data and interactive analytics to support knowledge-based effective community-based research and practice. Multi-faceted data have been classified as multi-dimensional, multi-variate, multi-modal, multi-run and multi-model [16]. Furthermore, interactive analytics is used, where a stored dataset can be queried in an ad-hoc manner in order to find useful information quickly [17]. We have applied visual analytics software to design and implement interactive analytics resources for use with or without access to internet-stored content [18,19,20].

The four objectives to help inform research and practice beneficial to stakeholders in sickle cell disease management are:

1. Construct datasets on textual data (sentences) in the sections of the RuSH Report on strategies for health promotion in sickle cell disease.
2. Design and implement interactive visual representations of textual data (sentences) in the sections of the RuSH Report on strategies for health promotion in sickle cell disease.
3. Determine themes in textual data on public health aspects and clinical aspects of sickle cell disease.
4. Design visual representations for comparing and integrating recommendations from expert reports on sickle cell disease in the United States.

## 2. Methods

### 2.1. Overview

Interactive analytics is used, where a stored dataset can be queried in an ad-hoc manner in order to find useful information quickly [17]. Interactive analytics involves human interaction with stored datasets through software such as those for constructing visualizations that support complex cognitive activities [20]. Complex cognitive activities include learning, decision-making, sense-making, knowledge discovery and planning [21]. The data to be interacted with are textual, where the textual structure of interest is the sentence (a combination of text strings), which is a textual unit of natural language processing applications [22] and a method of knowledge representation [23]. We also implement software generated word (tag) clouds as an interactive analytics technique [24].

### 2.2. Construction of Datasets

The Registry and Surveillance System for Hemoglobinopathies (RuSH) report on strategies for health promotion was the data source for constructing datasets on sentences associated with public health aspects of sickle cell disease. These sentences in the RuSH report constitute the content of the sections (Program Overview; Resources Needed; Benefits; Outcomes; and Lessons Learned) and were copied and stored in a spreadsheet file. Two datasets were constructed from the RuSH report: (1) the RuSH Strategies Dataset: Text Sentences from Sections of the Report; and (2) the RuSH Sites Dataset. The records in the datasets were annotated with record and sentence identifiers to facilitate integration of datasets. We constructed a dataset of titles of up to 100 scholarly publications citing the 2014 NHLBI Expert Panel Report on Evidence-Based Management of Sickle Cell Disease by searching the Google Scholar database. The title of the scholarly publication was selected since an informative title for a scholarly article will have “conveyed at least some general idea of the paper’s content, without recourse to other sources of information such as the abstract, the journal title, or the paper itself” [25].

The three expert reports on sickle cell disease (2014 NHLBI, 2019–2020 ASH, and 2020 NASEM) are the sources of textual data for constructing datasets of textual structures including words, phrases, sentences, and paragraphs [5,11,12,13,14,15]. For the 2014 NHLBI report, we constructed datasets of sentences from the text on key questions, associated recommendations (strong, moderate, weak, and consensus) and evidence (adapted, high-quality, moderate-quality, low-quality, very-low quality, and panel expertise). For the 2019–2020 ASH SCD guidelines, the questions, clinical practice, recommendation text, recommendations (conditional or strong) and associated evidence (high, moderate, low and very low) were used to construct datasets. For the NASEM SCD report, the document on the overview of the recommendations and strategic plan was the source for datasets including statements on recommendations and associated governmental agencies.

### 2.3. Development of Interactive Visual Representations

Visual representations can be in categories of plots and charts; maps; graphs, trees and networks; glyph and multidimensional icons; and enclosure diagrams [21]. The initial interactive visual representations developed were enclosure diagrams of the form of data tables or matrices. The interactive visual representations of the datasets were designed and implemented as views or dashboards in Tableau Desktop Professional (Tableau Inc. Washington, DC, USA) [26]. The collection of views and dashboards are available on the Tableau Public website.

Additionally, word clouds (or tag clouds) were constructed with software for sets of textual data (sentences) to compare the frequency of words between the datasets on public health aspects and clinical aspects of sickle cell disease. Word (tag) clouds, among other functions, facilitate rapid analysis of textual collections, knowledge integration, as well as meaningful and efficient ways of learning [27,28]. Word cloud is an interactive analytics technique [24].

## 3. Results and Discussion

### 3.1. Overview

Since the initial publication in 1910 of sickle shaped red blood cells, there have been thousands of scholarly publications on sickle cell disease. Textual data structures (such as symbols, words, phrases, sentences, paragraphs, figures, tables, documents and topics) from these scholarly publications are encoded in natural language and present prospects for constructing qualitative and quantitative datasets. We have constructed datasets and associated interactive visualizations to conduct interactive analytics of textual data structures on public aspects and clinical aspects of sickle cell disease. Our report presents a unique collection of data products that include landmark scholarly publications in the year 2020 from the American Society of Hematology and the National Academies of Science, Engineering and Medicine. The evidence or expert recommendations provide principles or guidelines for building a knowledgeable workforce for addressing sickle cell disease. Principle is one of the five types of knowledge that is a far-transfer task of decision making, where a professional has to consider the variables associated with the professional situation and decide on a solution to the task. The learning strategies of principles including simulations that allow for several perspectives to be tested by the learner. Thus, our interactive analytics computational resources for sickle cell disease guidelines has the potential to support lifelong learning through creativity and collaboration for continued development of individual and team professional expertise [29,30,31,32,33]. We present the results and discussion section to align with the four objectives.

### 3.2. Datasets and Interactive Visualizations on Health Promotion in Sickle Cell Disease

The sources of data were the Registry and Surveillance System for Hemoglobinopathies (RuSH) report and a collection of titles of journal articles citing the 2014 Evidence-Based Management of Sickle Cell Disease (NHLBI 2014 SCD report). The three datasets constructed from the two reports were in two categories: (1) RuSH report; and (2) Evidence-Based Management of Sickle Cell Disease (SCD) (Table 1, Table 2 and Table 3). There are eight sites in seven states: California (two RuSH sites), Florida, Georgia, Michigan, New York and North Carolina (Table 3). The themes of the health promotion activities of the hemoglobinopathy sites include: (1) focus group and stakeholder meetings; (2) online database resources; (3) provider network and directory; (4) community speaker panel; (5) health status assessment; (6) faith-based community outreach; and (7) a toll-free phone number for health care referrals.

The textual data structure (sentence) obtained from the report provides a knowledge representation that can be analyzed for knowledge content types (fact, concept, process, procedure and principle) [34] to provide deeper understanding for humans as well as computational processing. In the collection of seven sentences in Table 3, four sentences have the word “information” in the sentence, allowing us to have an integrated representation of the knowledge from the sentences from different sections of a site report.

In addition to the health promotion aspects, the RuSH project developed three-level case definitions and multiple data sets were used to collect information [35]. Our datasets on textual sentences of the RuSH health promotion report and titles of articles citing the NHLBI expert panel report is a novel contribution to integrating knowledge on public health aspects and clinical aspects of sickle cell disease. Online access to the datasets and visualizations are available as Supplementary Material. We previously constructed datasets and interactive visualizations to inform health promotion [18,20]. The interactive resources developed here allow users of the resources to explore scholarly publications for knowledge on the clinical and public health aspects of sickle cell disease.

The need to expand the understanding of the cause of sickle cell disease presents a significant and continuous need for new discoveries on all aspects of this hemoglobinopathy including clinical (individual) and public health (population) aspects. The public health significance of sickle cell disease is demonstrated by recent public law from the 115th United States Congress that amends the Public Health Service Act and is titled “Sickle Cell Disease and Other Heritable Blood Disorders Research, Surveillance, Prevention, and Treatment Act of 2018” (Public Health Law No. 115–327) [36,37].

**Figure 5:**
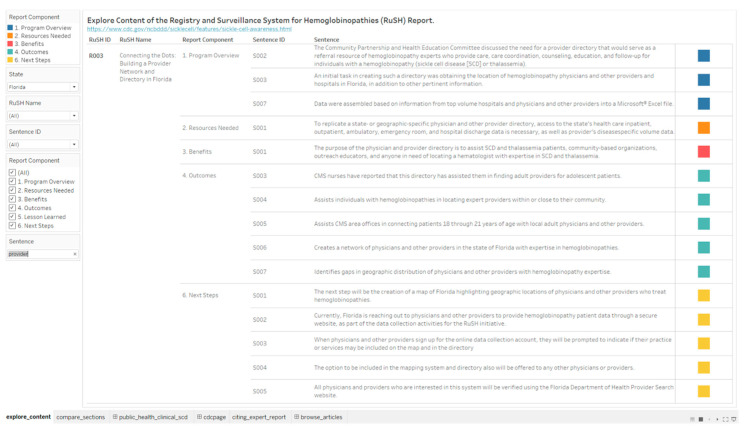
An interactive analytics enclosure table design for exploring textual data in the Registry and Surveillance System for Hemoglobinopathies (RuSH) Report.

During the surveillance period of 2004 to 2008, 39,633 individuals in Florida had a hemoglobinopathy diagnosis [35]. The RuSH site in Florida reported on building a provider network and directory (Figure 3 and Table 2). A search to retrieve sentences that contain the word “provider” retrieved 44 sentences from the reports contributed from six sites, with 15 associated with the RuSH site in Florida (Figure 5). The interactive analytics design includes a drop list menu (State, RuSH Name, Sentence ID and Report Component) for interacting with the underlying textual data. As part of the visualization, the sentences are color coded by the report section. The goals for visual analysis of data can be interaction, representation and analysis [16]. The options in the drop lists allow for modification of the interaction, visual representation and analysis.

The approaches of integrating datasets (such as visually-supported interactive analytics) from multiple aspects of sickle cell disease are increasingly needed for addressing surveillance and knowledge gaps on sickle cell disease in the United States and other countries [36,37]. The 2018 legislation on sickle cell disease in the United States includes the expansion of the Sickle Cell Data Collection (SCDC) system, a multi-data source, multi-organization, population-based, longitudinal system of the Centers for Disease Control and Prevention (CDC) [37]. Thus, there is an anticipated significant increase in public health datasets on sickle cell disease that must be transformed into knowledge for diverse applications.

The SCDC data resources could also provide real world datasets for public health data science education. Research and learning activities in undergraduate and graduate programs on public health can be guided by frameworks for data science challenges. The dimensions of data science challenges according to the National Data Science Consortium are Data Flow (access, collection, movement and storage), Data Curation (cleaning, description, preservation, publication, and security), and Data Analytics (modeling and simulation, statistical analysis, and visual analytics) [38]. The interactive analytics here are implemented with visual analytics tools.

Since the 2014 NHLBI guidelines on management of sickle cell disease, according to researchers of the OneFlorida Clinical Data Research Network (CDRN), there has been a slight, but statistically significant, increase in Hydroxyurea Utilization (HU) for the treatment of sickle cell anemia in Florida [39]. The research study also identified 13 comprehensive sickle cell centers based on “(1) local expert opinion, (2) review of the American Society of Hematology Find a Hematologist database, and (3) involvement in recent multicenter clinical trials.”

### 3.3. Themes in Textual Data on Public Health and Clinical Aspects of Sickle Cell Disease

**Figure 6:**
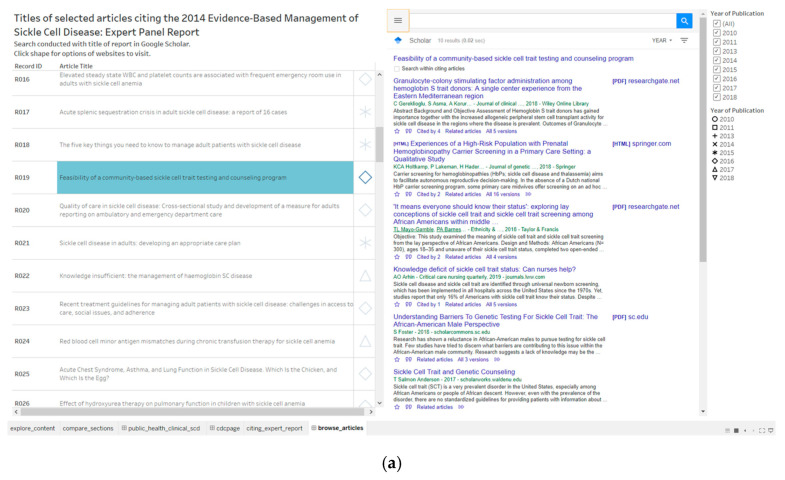

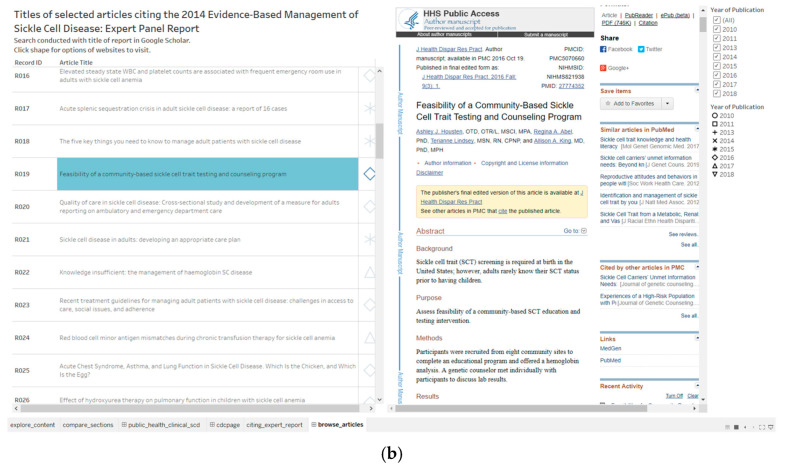
A dashboard combines an enclosure table and a website to browse articles on public health and clinical aspects of sickle cell disease. (**a**) Result of a Google Scholar search in the dashboard; (**b**) display of journal article web page.

We have collected 99 journal articles that cited the 2014 NHLBI expert panel report on evidence-based management of sickle cell disease. We have constructed a dashboard to connect to the web page of the citing journal article in Google Scholar as well as the journal article in the publisher’s or full text web page (Figure 6). This connection to scholarly communication resources ensures that the interactive analytics resources developed have continuous relevance for tracking public health and clinical aspects that cite the NHLBI expert panel report on sickle cell disease management. An example citing article published in 2016 has the title “Feasibility of a community-based sickle cell trait testing and counseling program”. The citing articles subsequently citing the feasibility study include at least two that have a focus on screening for sickle cell trait in African Americans (Figure 6a). The clinical and public health implications of the sickle cell trait continues to be relevant in the United States [40]. For example, sickle cell trait is associated with chronic kidney disease [41,42].

The titles of other public health related articles citing the NHLBI expert panel report are: (1) “Recent treatment guidelines for managing adults patients with sickle cell disease: challenges in access to care, social issues, and adherence; and, (2) “Sickle cell disease in adults: developing an appropriate care plan”. Examples of clinical aspects retrieved are: (1) “Acute splenic sequestration crisis in adult sickle cell disease: a report of 16 cases”; (2) “Red blood cell minor antigen mismatches during chronic transfusion therapy for sickle cell anemia”; and, (3) “Effect of hydroxyurea therapy on pulmonary function in children with sickle cell anemia”. The topic of treatment and care is a topic of clinical and public health aspects of sickle cell disease. An integrated perspective is home-based care of sickle cell disease, especially in circumstances that prevent out-patient primary care [43]. These scholarly publications contain knowledge learning objects for recycling knowledge [44] and generating new knowledge [45]. Our interactive analytics approach provides an environment to interact with the knowledge learning objects on sickle cell disease and support the performance of learning and other complex cognitive activities [21].

**Figure 7:**
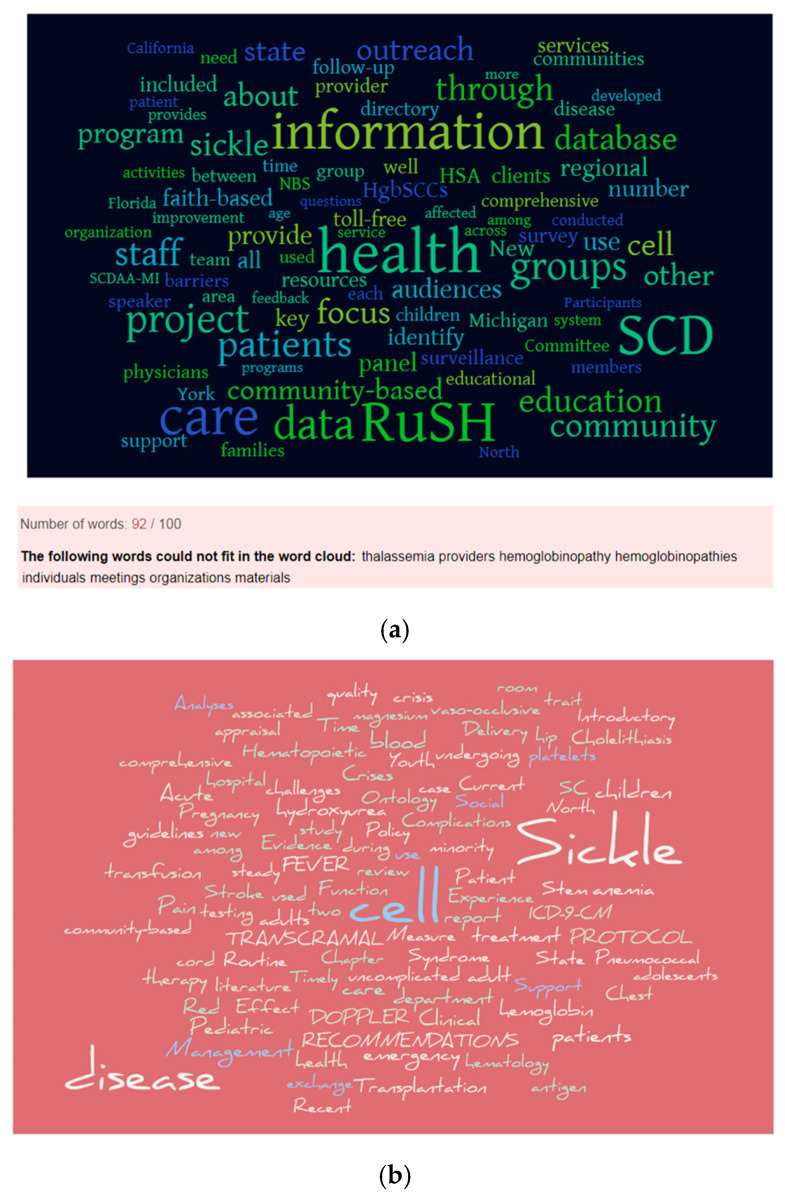
A comparison of word clouds generated from textual data on public health and clinical aspects of sickle cell disease. A common term to both aspects is “community-based”. (**a**) A word cloud of textual sentences from sections of the Registry and Surveillance System for Hemoglobinopathies (RuSH) report; (**b**) a word cloud of the titles of journal articles citing the National Heart, Lung and Blood Institute (NHLBI) Sickle Cell Disease Expert Panel report.

We compared the text extracted from the RuSH report to the titles of the journal articles citing the NHLBI expert panel report (Figure 7). The words identified can be further analyzed into groups depending on the public health or clinical aspects of interest to a professional. Some public health relevant words from the RuSH word cloud are barriers, faith-based, outreach, services, and surveillance. The RuSH word cloud reveals the word “information” among the top occurring words along with worlds health, RuSH and SCD. Information, along with data, evidence and knowledge, is a central concept in health informatics and data science [46]. According to the Data–Information–Evidence–Knowledge (DIEK) framework: “data are raw symbols, which become information when they are contextualized. Information achieves the status of evidence in comparison to relevant standards.” Furthermore, “evidence is used to test hypotheses and is transformed into knowledge by success and consensus”. The DIEK framework has relevance to all aspects of sickle cell disease.

The word cloud for the journal articles citing the NHLBI report presents words describing clinical aspects such as treatment approaches (e.g., hydroxyurea, magnesium, transfusion, and transplantation), clinical manifestations (e.g., crisis, cholelithiasis, pain, pneumococcal, vaso-occlusive, and stroke), population (e.g., adolescents, adult, minority, pediatric, pregnancy, and youth), and human body structures (e.g., antigen, blood, chest, cord, hemoglobin, hip, platelets). The multiple population groups identified in the word cloud of titles citing the NHLBI expert panel report allude to the lifelong clinical aspects of sickle cell disease [6]. The population groups also present the need for research and practice on the public health aspects directed to specific populations. In particular, a globally recognized public health need is the effective pediatric to adult transition care for patients with sickle cell disease [47,48].

In April 2020, a multi-country task force (the USA, Europe, Middle East, and Africa) recommended a process of transition in this high risk period that includes Skills transfer, Increasing self-efficacy, Coordination, Knowledge transfer, Linking to adult services, and Evaluating readiness (the SICKLE recommendations) [48]. The SICKLE recommendations present opportunities to use interactive visualizations for the knowledge transfer on sickle cell disease knowledge content types to patients transitioning from pediatric to adult care. Among the knowledge items used to assess patient readiness for transition are: (1) name and type of the disease; (2) the main genetic and pathophysiological characteristics; (3) baseline hemoglobin level; (4) treatment, role of each drug; (5) adherence to treatment; (6) course of action in the event of pain, fever, or priapism; and (6) method for escalating analgesics [49]. We recently advocated for the use of knowledge visualization frameworks in the creation and transfer of complex public health knowledge [18]. Sickle cell disease is an example of complex public health knowledge, where effective, efficient and engaging (i.e., smarter) learning environments [50] on a large and sustainable scale are urgently needed in an era of social distancing due to the COVID-19 pandemic [51]. Furthermore, COVID-19 has led to the expansion of remotely supported access to health care (such as telemedicine, telehealth, ehealth, mhealth, virtual reality, augmented reality, remote treatment or therapy and digital therapeutics) [52]. The expert recommendations can be integrated within these remote digital platforms to expand access to care for individuals with sickle cell disease. Furthermore, the integrated datasets from sickle cell disease registries, such as Surveillance Epidemiology of Coronavirus (COVID-19) Under Research Exclusion [53,54], can enhance understanding of sickle cell disease.

We note that “community-based” is common to both the RuSH report and the NHLBI expert panel report. The advances in the past decade (2011 to 2020) on the therapy of sickle cell disease include: (1) modifying the patient’s genotype; (2) targeting hemoglobin S polymerization; (3) targeting vasocclusion; and (4) targeting inflammation [55]. These advances in genome science and technology have led to potential therapy for sickle cell disease using CRISPR genome editing technology. New genome-based technology for developing therapies presents opportunities for community-based participatory research [56,57,58].

### 3.4. Designs of Visual Representations for Comparing and Integrating Recommendations from Expert Reports on Sickle Cell Disease in the United States

**Figure 8:**
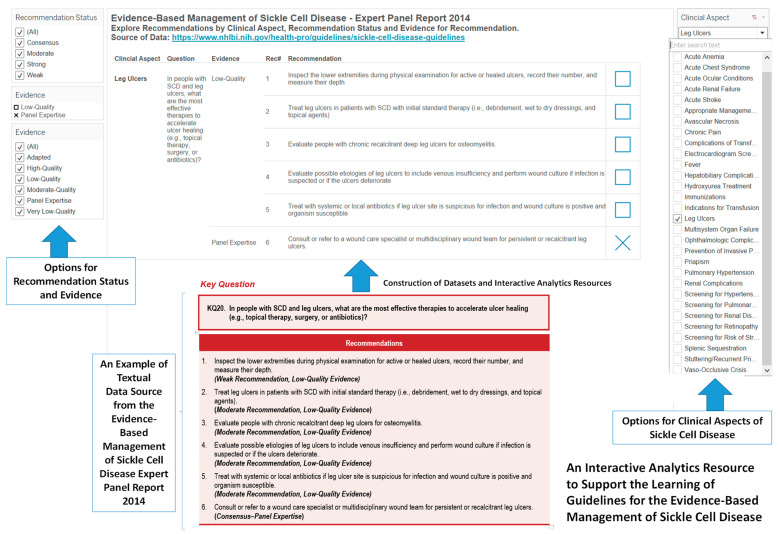
An example design of an interactive analytics to support the learning of guidelines for the evidence-based management of sickle cell disease. In this example, the key question and recommendations for evidence-based management of leg ulcers is included to show the textual data source.

The expert reports on sickle cell disease are resources for understanding the diverse aspects of sickle cell disease. Thus, we have constructed example designs of interactive analytics resources to support the outcomes of robust learning (long-term retention, transfer of learning and desire for future learning) of the diverse aspects of sickle cell disease in the evidence-based management of sickle cell disease expert panel report, 2014 (Figure 8). Learning requires interaction and navigation, thus presenting knowledge on sickle cell disease in interactive analytics that can be modified by learner will help promote robust learning, especially in the increasing circumstances of remote learning.

**Figure 9:**
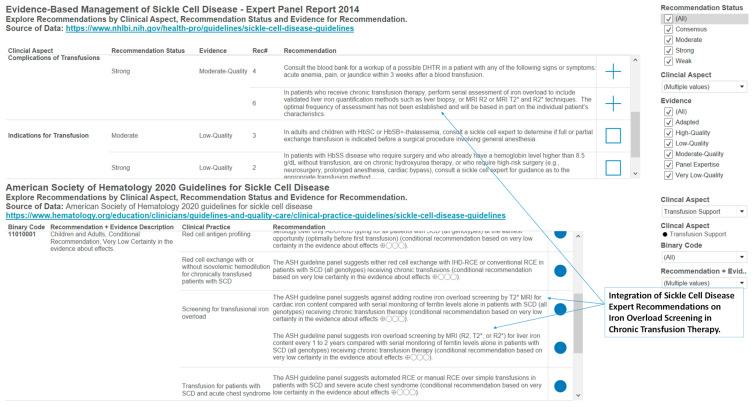
An example design of an interactive visual representation that allows for integrating and comparing expert recommendations on clinical aspects of sickle cell disease. In this example, we highlight the integration of sickle cell disease expert recommendations on iron overload screening in chronic transfusion therapy.

The availability of multiple expert recommendations also presents the need for resources to support comparisons and integration of recommendations. Therefore, we provide an example dashboard design to support the comparison and integration of recommendations from expert panels. In Figure 9, the dashboard design supports comparison and integration of recommendations on blood transfusion in sickle cell disease from guidelines in the 2014 NHLBI SCD report and the 2019–2020 ASH reports. A finding from this dashboard (Figure 9) is that in the case of sickle cell patients receiving chronic transfusion therapy, the 2019–2020 ASH guideline suggests the frequency of iron overload screening for liver iron content to be every 1 to 2 years, while the 2014 NHLBI guideline states that “the optimal frequency of assessment has not been established and will be based on the individual patient’s characteristics.” The dashboard combines the two clinical practice guidelines and can also support the recognition of differences that can be a basis for inquiry and investigation.

For within expert report comparison and integration, we constructed binary codes to represent presence (1) or absence (0) of features. In the 2020 ASH guidelines, we applied an eight-digit binary number to encode the patient group (digit 1 and 2: children, adult), strength of recommendation (digit 3 and 4: strong, conditional) and evidence about effects (digits 5, 6, 7 and 8: high, moderate, low and very low). Therefore, it is possible to group recommendations from multiple clinical aspects of sickle cell disease by an eight-digit binary number. We have previously used the binary number encoding for integrating data on farmers markets as well as obesity rates [18,20].

**Figure 10:**
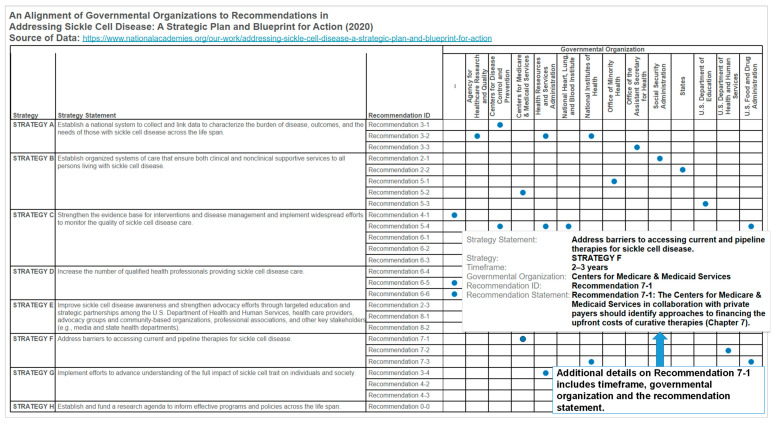
An example design of an interactive visual representation that allows for integrating and comparing strategies and expert recommendations on clinical and public health aspects of sickle cell disease. In this example, we highlight the alignment of governmental organizations to strategies such as financing the upfront costs of curative therapies by the Centers for Medicare & Medicaid Services. The interactive analytics resource could function as a decision aid for identifying potential funding sources for sickle cell disease initiatives in the United States.

We constructed datasets and interactive analytics from the overview document of the 2020 National Academies of Science, Engineering and Medicine (NASEM) consensus report “Addressing Sickle Cell Disease: A Strategic Plan and Blueprint for Action” [15,59]. An example interactive analytics (Figure 10) allows for the comparison and integration of the eight strategies, associated recommendations and the governmental organizations. Through the interactive enclosure table visual (Figure 10), a stakeholder could explore the strategies and recommendations on the clinical and public health aspects of sickle cell disease. One of the 2020 NASEM strategies is “increase the number of qualified health professionals providing sickle cell disease care” and includes a recommendation for health professional organizations to support sickle cell disease providers through education, credentialing, networking, and advocacy. The education and credentialing efforts will require designing learning experiences for the complex knowledge content in expert reports and scholarly articles on sickle cell disease. An approach is to apply the framework of the five knowledge content types: facts, concepts, processes, procedures and principles [34]. In particular, the learning of the expert recommendations or guidelines can use suggested activities for learning principles such as analogies, simulations, and role play [60]. The implementation of the 2020 NASEM sickle cell disease strategies will require a new cadre of professionals who are able to perform data-related duties and tasks and participate in a sickle cell disease “research agenda to inform effective programs and policies across the life span.”

Our current report presents integrated datasets on clinical and public health aspects of sickle cell disease. We also acknowledge the availability of other reports that contain content that validates the accuracy of the content displayed by our interactive analytics. For example, in March 2020, the Institute for Clinical and Economic Review (ICER) published a 282-page evidence report on the comparative clinical effectiveness and value of three pharmaceutical agents (crizanlizumab, voxelotor, and L-glutamine) for the treatment of sickle cell disease [61]. This first ICER report on sickle cell disease lists some recommendations from the 2014 NHLBI and 2020 ASH guidelines. The 2014 NHLBI report recommendations in the 2020 ICER report are those labeled as strong for clinical aspects of acute pain crisis, acute chest syndrome, acute and chronic transfusion, hemoglobin, hydroxyurea, and stroke.

**Figure 11:**
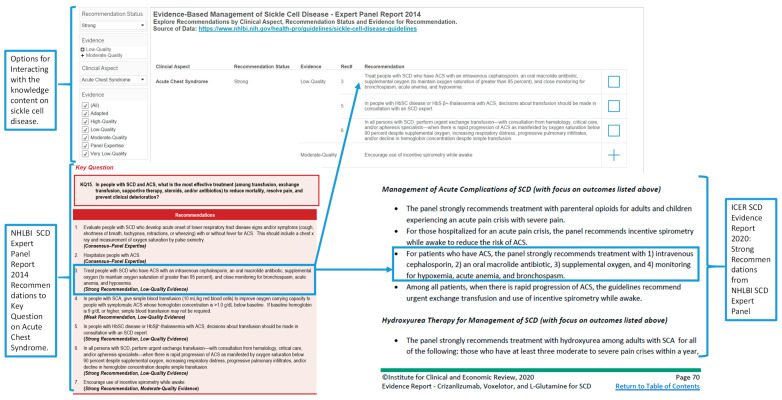
A validation of content of the interactive analytics resource for exploring recommendations on evidence-based management of sickle cell disease. The interactive analytics resource enables interactions to support decision making on the clinical aspects of sickle cell disease and associated recommendations and evidence. The example is for acute chest syndrome, one of the most common and serious complications of sickle cell disease.

The list of recommendations in the ICER report provided an independent list to determine whether our interactive analytics resource for the 2014 NHLBI report will retrieve the same subset of strong recommendations presented in the 2020 ICER report. Our interactive analytics resource retrieved the same strong recommendations as the 2020 ICER report (Figure 11). The 2020 ICER report on sickle cell disease contains textual and tabular data structures suitable for interactive analytics to support learning and other complex cognitive activities. For example, an appendix with ongoing clinical trials contains expert-compiled contents: (1) Title/Trial Sponsor; (2) Study Design; (3) Study Arms; (4) Patient Population; (5) Primary Outcomes Estimated; and (6) Completion Dates. Future research could investigate designs of interactive analytics that can promote the desire for future learning and participation in clinical trials by community stakeholders.

### 3.5. Study Limitations

Our study could have the general limitations associated with secondary data analysis. For example, the purpose of the initial data collection can be different to our study. For the 2020 NASEM and 2019–2020 ASH report, we implemented our approach on selected sections as demonstration of designs. Future investigations could expand the textual data selected from these expert/evidence/consensus reports to answer specific questions. Despite the potential limitations, our approach of integrating datasets through interactive analytics has led to several findings including focus areas for community-based research and practice on sickle cell disease. Additionally, we have designed interactive analytics resources that connect continuously updated online scholarly databases. We also confirmed the consistency of the textual data in the interactive analytics with textual data in expert or evidence reports.

## 4. Conclusions

Sickle cell disease (SCD) is a genetic disease that has multiple aspects including public health and clinical aspects. The goals of the research study were to (1) understand the public health aspects of sickle cell disease and (2) understand the overlap between public health aspects and clinical aspects that can inform research and practice beneficial to stakeholders in sickle cell disease. We have constructed datasets from textual data sources produced by experts on sickle cell disease. We have designed interactive analytics to display the expert recommendations on clinical and public health aspects. The interactive analytics allows for comparisons and integrations of expert recommendations. Our report is timely and current on sickle cell disease in the United States. We have used interactive visualizations to integrate datasets in evidence and expert reports including those published in 2019 and 2020.

A major finding from our study is the global public health need for effective pediatric to adult transition care for patients with sickle cell disease. We propose that interactive analytics of textual data structures can support the knowledge transfer stage, including the transitioning care programming, in patients with sickle cell disease. Technology-supported learning systems are now especially relevant in the time of social distancing due to the 2019 coronavirus disease (COVID-19) pandemic. Another major finding reported here is that community-based approaches are of interest to both public health and clinical aspects of sickle cell disease. We propose that advances in genome science and technologies (such as CRISPR genome editing) for developing therapies for sickle cell disease present opportunities for community-based participatory research.

Considering the multifaceted datasets on sickle cell available through scholarly publications, we propose that data structures (i.e., text, figures, figure captions, data tables, and Supplementary Materials) in scholarly publications on sickle cell disease encode (1) knowledge content types (fact, concept, process, procedure and principles) and (2) cognitive content (data, information, evidence and knowledge) for interactive analytics to recycle and generate new knowledge on sickle cell disease.

## Figures and Tables

**Table 1:** Datasets constructed for understanding public and clinical aspects of sickle cell disease.

Dataset Name ^1^	Dataset Features
RuSH Strategies Dataset: Text Sentences from Sections of Report	225 Sentences, Six sections of RuSH report, 11 categories of sentences based on location in the section.
RuSH Sites Dataset: Sites	8 RuSH sites, 7 States.
Evidence-Based Management of SCD: Titles	99 articles, 49 Google Scholar IDs and 99 Article Identifiers.

^1^ RuSH: Registry and Surveillance System for Hemoglobinopathies; SCD: Sickle Cell Disease.

**Table 2:** Sites for the Registry and Surveillance System for Hemoglobinopathies (RuSH) Report.

RuSH ID	RuSH Name	State
R001	Putting the Focus on Sickle Cell: Northern California’s Focus Group and Stakeholder Meetings	California
R002	Resource STREET: Sickle Cell Disease and Thalassemia Resources to Educate and Empower the Community—An Online Database Serving Southern California	California
R003	Connecting the Dots: Building a Provider Network and Directory in Florida	Florida
R004	Finding Our Voice: Creating and Implementing a Community Speaker Panel in Georgia	Georgia
R005	Measuring Michigan’s Health: A Hemoglobinopathy Health Status Assessment	Michigan
R006	Garnering State-wide Support for RuSH: Regional Provider Meetings in New York	New York
R007	Faith-based Initiative: North Carolina’s Approach to Community Outreach for Hemoglobinopathies	North Carolina
R008	Answering the Call: Pennsylvania’s Toll-free Phone Number for Hemoglobinopathy Health Care Referrals	Pennsylvania

**Table 3:** Example of sentences in sections of the Registry and Surveillance System for Hemoglobinopathies (RuSH) Report.

RuSH ID.	Report Component	Sentence ID	Sentence ^1^
R001	Program Overview	S001	Individuals with hemoglobinopathies, specifically sickle cell disease (SCD) and thalassemia, face significant barriers accessing health care services and participating in public health and clinical research projects.
R001	Lessons Learned	S001	15 min should be allotted for general education on hemoglobinopathies at the beginning of the focus groups, especially for groups that are further removed from health care fields, such as cultural organizations, sororities, and fraternities.
R002	Resources Needed	S001	The fundamental computer code developed for the database is available to anyone interested in building a database with similar components.
R002	Benefits	S001	There are significant benefits to having **information** in one place, immediately accessible to users, and flexible so that users can enter the **information** and create personalized groups.
R002	Outcomes	S001	The database has been invaluable for generating mailing lists to send outreach materials, surveys, and other **information** to target groups of providers and organizations.
R002	Lessons Learned	S001	Database development is a trial and error process, and it is difficult to anticipate exactly how long each stage of development will take.
R002	Next Steps	S001	This database serves as a common repository of **information** to identify providers, agencies, and individuals with resources for SCD and thalassemia.

^1^ A common word, “information”, is highlighted in bold.

## Supplementary Materials

The online versions of the interactive analytics resources produced are available at https://public.tableau.com/profile/qeubic#!/vizhome/scd_text_analytics001/overview.
